# Age and Prolonged Work Absence After Occupational Same‐Level Fall Injuries in Japan: A Nationwide Retrospective Study

**DOI:** 10.1111/ggi.70662

**Published:** 2026-07-10

**Authors:** Ryutaro Matsugaki, Hajime Ando, Akira Ogami

**Affiliations:** ^1^ Department of Work Systems and Health Institute of Industrial Ecological Sciences, University of Occupational and Environmental Health Kitakyushu Japan

## Abstract

This nationwide retrospective study using the Japanese National Lost‐Time Occupational Injury Microdata demonstrated that older workers experience longer work absences after same‐level fall injuries than younger workers. These findings highlight the need for age‐specific prevention and return‐to‐work support, particularly for older workers.

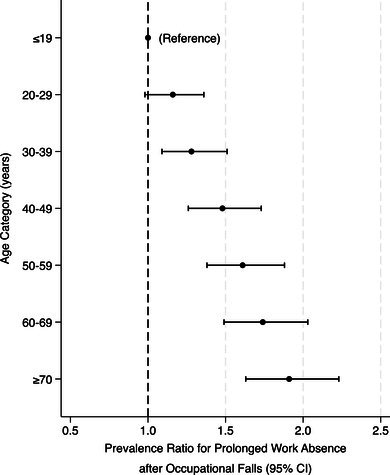

Dear Editor,

1

Same‐level falls are among the most common occupational injuries worldwide [1, 2]. In addition to their impact on workers' health, same‐level fall injuries impose a substantial socioeconomic burden due to work absences. In Japan, the government has prioritized preventing same‐level falls and reducing post‐fall work absence duration as key occupational health objectives [3]. While international studies suggest that older workers experience longer absences from work after occupational injuries than younger workers [4, 5], it remains unclear whether this applies specifically to same‐level fall injuries. Clarifying this is crucial to determining whether interventions to reduce prolonged absences after same‐level falls should be applied uniformly across all age groups or targeted specifically at older workers. Using the Japanese National Lost‐Time Occupational Injury Microdata (J‐LTOI), which includes all occupational injuries with ≥ 4 days of work absence reported to Labor Standards Inspection Offices [6], this study examined the association between age and prolonged work absence after same‐level falls. This study provides foundational data for evidence‐based intervention strategies.

This retrospective observational study analyzed 2023 J‐LTOI data. It included 36 023 nonfatal same‐level fall cases among workers in all age groups. Age was categorized into seven groups: ≤ 19, 20–29, 30–39, 40–49, 50–59, 60–69, and ≥ 70 years. Prolonged work absence was defined as ≥ 1 month based on J‐LTOI's estimated duration categories, aligning with international standards and Japan's 14th Occupational Safety and Health Program target [3, 7]. A modified Poisson regression with robust error variance was used to estimate the prevalence ratios (PRs) and 95% confidence intervals (CIs) [8], adjusting for sex, industry sector, establishment size, season, and region. As a supplementary analysis, we first tested for an interaction between age category and fracture status. We then conducted analyses stratified by fracture versus non‐fracture injury to examine whether the association between age and prolonged work absence differed according to injury characteristics. As a sensitivity analysis for unmeasured confounding, E‐values were computed for both point estimates and lower 95% CIs limits of the PRs using the *evalue* command in Stata [9, 10]. Statistical significance was set at *p* < 0.05 (Stata 18.0, StataCorp, TX).

Among 36 023 same‐level fall cases (40.1% male), prolonged work absence (≥ 1 month) occurred in 59.4% (21 400 cases). The proportion of prolonged absences increased with age: 36.8% (≤ 19 years), 42.1% (20–29 years), 46.8% (30–39 years), 53.9% (40–49 years), 58.6% (50–59 years), 63.7% (60–69 years), and 70.2% (≥ 70 years). After adjusting for covariates, risk of prolonged absence progressively increased with age compared to workers aged ≤ 19 years (reference group): PRs 1.16 (95% CI: 0.98–1.36, *p* = 0.085) for 20–29 years; 1.28 (95% CI: 1.09–1.51, *p* = 0.002) for 30–39 years; 1.48 (95% CI: 1.26–1.73, *p* < 0.001) for 40–49 years; 1.61 (95% CI: 1.38–1.88, *p* < 0.001) for 50–59 years; 1.74 (95% CI: 1.49–2.04, *p* < 0.001) for 60–69 years; and 1.91 (95% CI: 1.63–2.23, *p* < 0.001) for ≥ 70 years (*p* for trend < 0.001) (Table 1). A statistically significant interaction between age category and fracture status was detected (*p* for interaction = 0.005), with higher prevalence ratios observed in the non‐fracture group (PR for ≥ 70 years: 1.62) than in the fracture group (PR for ≥ 70 years: 1.36) (Table S1). E‐value sensitivity analyses for unmeasured confounding are presented in Table S2. The *E*‐values for the prevalence ratios ranged from 1.88 to 3.23 for point estimates and from 1.40 to 2.64 for the lower 95% confidence limits across age categories.

**TABLE 1:** Association between age and prolonged work absence after occupational falls.

	Prolonged work absence after occupational same‐level falls	Crude model				Multivariate model*			
		PRs	95% CI	*p*	*p* for trend	PRs	95% CI	*p*	*p* for trend
Age, years									
≤ 19 (*n* = 272)	36.8% (100/272)	Reference				Reference			
20–29 (*n* = 1885)	42.1% (794/1885)	1.15	(0.97–1.35)	0.105	< 0.001	1.16	(0.98–1.36)	0.085	< 0.001
30–39 (*n* = 2370)	46.8% (1109/2370)	1.27	(1.08–1.50)	0.003		1.28	(1.09–1.51)	0.002	
40–49 (*n* = 4888)	53.9% (2633/4888)	1.47	(1.25–1.72)	< 0.001		1.48	(1.26–1.73)	< 0.001	
50–59 (*n* = 10 378)	58.6% (6086/10 378)	1.60	(1.36–1.87)	< 0.001		1.61	(1.38–1.88)	< 0.001	
60–69 (*n* = 10 924)	63.7% (6954/10 924)	1.73	(1.48–2.02)	< 0.001		1.74	(1.49–2.04)	< 0.001	
70 ≥ (*n* = 5306)	70.2% (3724/5306)	1.91	(1.63–2.23)	< 0.001		1.91	(1.63–2.23)	< 0.001	

*Note:* Multivariate model: Adjusted for sex, industry classification, establishment size, season, and area.

Abbreviations: 95% CI: 95% confidence interval; PRs, prevalence ratios.

* Indicates that the multivariate model was adjusted for sex, industry classification, establishment size, season, and area.

These findings demonstrate that international evidence suggesting longer work absences after occupational injuries among older workers compared to younger workers also applies to same‐level falls among Japanese workers [4, 5]. Of note, although this association was observed in both fracture and non‐fracture subgroups, a statistically significant interaction between age and fracture status was detected (*p* for interaction = 0.005), suggesting that the magnitude of the association between age and prolonged work absence differed by fracture status. The attenuation of the age effect in the fracture group may reflect the dominant influence of fracture severity on recovery time, which might reduce the relative contribution of age. In contrast, among workers without fractures, age‐related declines in recovery capacity and accumulation of comorbidities, as well as difficulties in workplace return‐to‐work adjustment due to the absence of objective clinical criteria for recovery, may play a larger role in prolonging work absence. Therefore, preventing prolonged work absence requires not only a primary prevention focus on fall and fracture prevention, but also tertiary prevention, including rehabilitation and workplace support aimed at facilitating a return to work, particularly for older workers.

A strength of this study is its use of comprehensive national data on all occupational injuries with ≥ 4 days of absence from work. However, the study had several limitations. First, cases with < 4 days of lost work were excluded, which may have affected the results. Second, unmeasured confounding factors, such as workers' comorbidities and workplace return‐to‐work support systems, as well as workers' baseline health status, physical functioning, and quantitative measures of occupational physical workload, may have affected the observed associations. However, *E*‐value sensitivity analyses indicated that a full accounting for the observed age effect for workers aged 70 years and older, who showed the strongest association, would require an unmeasured confounder to be associated with both age and prolonged work absence by a prevalence ratio of at least 2.64 (*E*‐value for the lower 95% confidence limit; corresponding *E*‐value for the point estimate, 3.23), above and beyond the measured confounders. This in turn suggests that the observed associations are robust to a substantial degree of unmeasured confounding. Finally, the study could not elucidate the mechanisms underlying long‐term absenteeism among older workers. Understanding these mechanisms is imperative for developing strategies to reduce long‐term absenteeism, representing a critical area for future research.

In conclusion, aging was associated with prolonged work absence following same‐level falls. Older workers had a higher prevalence of prolonged work absence than younger workers. Therefore, it is necessary to develop countermeasures focused on older workers to shorten the duration of absence from work following same‐level fall injuries.

## Author Contributions

**Ryutaro Matsugaki:** conceptualization, methodology, formal analysis, investigation, writing – original draft, project administration, funding acquisition. **Hajime Ando:** writing – review and editing. **Akira Ogami:** writing – review and editing.

## Funding

This work was supported by the Japan Society for the Promotion of Science (grant number 24K20394).

## Ethics Statement

Ethical approval was not required for this study because the J‐LTOI data were anonymized and are publicly available.

## Conflicts of Interest

The authors declare no conflicts of interest.

## Supporting information


**Table S1:** Association between age and prolonged work absence stratified by fracture versus non‐fracture injury.

**Table S2:** *E*‐values for the association between age and prolonged work absence after occupational fall.

## Data Availability

The data that support the findings of this study are available from the corresponding author upon reasonable request.
