# Supplementary material for: Age and Prolonged Work Absence After Occupational Same‐Level Fall Injuries in Japan: A Nationwide Retrospective Study
Source: Geriatr Gerontol Int. 2026 Jul 10;26(7):e70662. doi: 10.1111/ggi.70662 (PMC13354719; doi:10.1111/ggi.70662)
Supplement: Supplementary file 1 — Table S1: Association between age and prolonged work absence stratified by fracture versus non‐fracture injury. [file GGI-26-0-s001.pdf]

Supplementary Table S1. Association between age and prolonged work absence stratified by fracture versus non-fracture injury

	Prolonged work absence after occupational same-level falls	Crude model				Multivariate model*			
		PRs	95%CI	p-value	p for trend	PRs	95%CI	p-value	p for trend
Without fracture (n=12,185)									
Age, years									
≤19 (n=176)	23.3% (41/176)	Reference				Reference			
20-29 (n=1051)	25.5% (268/1051)	1.09	(0.82-1.46)	0.537	<0.001	1.09	(0.82-1.46)	0.557	<0.001
30-39 (n=1052)	23.6% (248/1052)	1.01	(0.76-1.35)	0.936		1.01	(0.75-1.35)	0.959	
40-49 (n=1927)	30.3% (584/1927)	1.30	(0.99-1.72)	0.062		1.32	(1.00-1.75)	0.050	
50-59 (n=3350)	29.4% (986/3350)	1.26	(0.96-1.66)	0.093		1.32	(1.00-1.74)	0.048	
60-69 (n=3128)	30.3% (948/3128)	1.30	(0.99-1.71)	0.059		1.36	(1.03-1.79)	0.030	
≥70 (n=1501)	37.6% (564/1501)	1.61	(1.22-2.12)	0.001		1.62	(1.22-2.13)	0.001	
With fracture (n=23,838)									
Age, years									
≤19 (n=96)	61.5% (59/96)	Reference				Reference			
20-29 (n=834)	63.1% (526/834)	1.03	(0.89-1.21)	0.761	<0.001	1.04	(0.88-1.23)	0.628	<0.001
30-39 (n=1318)	65.3% (861/1318)	1.06	(0.90-1.25)	0.464		1.08	(0.92-1.27)	345	
40-49 (n=2961)	69.2% (2049/2961)	1.13	(0.96-1.32)	0.147		1.14	(0.97-1.34)	0.103	
50-59 (n=7028)	72.6% (5100/7028)	1.18	(1.01-1.38)	0.041		1.19	(1.02-1.40)	0.029	
60-69 (n=7796)	77.0% (6006/7796)	1.25	(1.07-1.47)	0.005		1.27	(1.08-1.48)	0.004	
≥70 (n=3805)	83.1% (3160/3805)	1.35	(1.15-1.58)	<0.001		1.36	(1.16-1.60)	<0.001	

Multivariate model: Adjusted for sex, industry classification, establishment size, season, and area. PRs: prevalence ratios, 95%CI: 95% confidence interval.
