# Supplementary material for: Age and Prolonged Work Absence After Occupational Same‐Level Fall Injuries in Japan: A Nationwide Retrospective Study
Source: Geriatr Gerontol Int. 2026 Jul 10;26(7):e70662. doi: 10.1111/ggi.70662 (PMC13354719; doi:10.1111/ggi.70662)
Supplement: Supplementary file 2 — Table S2: E‐values for the association between age and prolonged work absence after occupational fall. [file GGI-26-0-s002.pdf]

Supplementary Table S2. E-values for the association between age and prolonged work absence after occupational same-level falls.

Age, years	PRs	95% CI	E-value	
			Point Estimate	Lower 95% CI
≤19	Reference	—	—	—
20–29	1.16	0.98–1.36	—	—
30–39	1.28	1.09–1.51	1.88	1.40
40–49	1.48	1.26–1.73	2.32	1.83
50–59	1.61	1.38–1.88	2.60	2.10
60–69	1.74	1.49–2.03	2.88	2.34
≥70	1.91	1.63–2.23	3.23	2.64

PRs: prevalence ratios, 95%CI: 95% confidence interval.
